# Identification of responsive gene modules by network-based gene clustering and extending: application to inflammation and angiogenesis

**DOI:** 10.1186/1752-0509-4-47

**Published:** 2010-04-21

**Authors:** Jin Gu, Yang Chen, Shao Li, Yanda Li

**Affiliations:** 1 MOE Key Laboratory of Bioinformatics and Bioinformatics Division, Tsinghua National Laboratory for Information Science and Technology (TNLIST) and Department of Automation, Tsinghua University, Beijing 100084, China

## Abstract

**Background:**

Cell responses to environmental stimuli are usually organized as relatively separate responsive gene modules at the molecular level. Identification of responsive gene modules rather than individual differentially expressed (DE) genes will provide important information about the underlying molecular mechanisms. Most of current methods formulate module identification as an optimization problem: find the active sub-networks in the genome-wide gene network by maximizing the objective function considering the gene differential expression and/or the gene-gene co-expression information. Here we presented a new formulation of this task: a group of closely-connected and co-expressed DE genes in the gene network are regarded as the signatures of the underlying responsive gene modules; the modules can be identified by finding the signatures and then recovering the "missing parts" by adding the intermediate genes that connect the DE genes in the gene network.

**Results:**

ClustEx, a two-step method based on the new formulation, was developed and applied to identify the responsive gene modules of human umbilical vein endothelial cells (HUVECs) in inflammation and angiogenesis models by integrating the time-course microarray data and genome-wide PPI data. It shows better performance than several available module identification tools by testing on the reference responsive gene sets. Gene set analysis of KEGG pathways, GO terms and microRNAs (miRNAs) target gene sets further supports the ClustEx predictions.

**Conclusion:**

Taking the closely-connected and co-expressed DE genes in the condition-specific gene network as the signatures of the underlying responsive gene modules provides a new strategy to solve the module identification problem. The identified responsive gene modules of HUVECs and the corresponding enriched pathways/miRNAs provide useful resources for understanding the inflammatory and angiogenic responses of vascular systems.

## Background

Understanding of cell responses to environmental stimuli is one of the central tasks of molecular biology. Genome-wide gene expression profiling techniques, such as microarray and deep sequencing, are widely used to identify the responsive genes whose expressions are significantly changed after the stimulus. But identifying the responsive genes by differential expressions does not consider the complex gene-gene interactions or regulation information. Increasing evidences suggest that cell responses are usually organized as pathways or responsive gene modules consisting of a group of interacted genes at the molecular level [1-4]. Identification of the responsive gene modules rather than independent responsive genes can provide better understanding of the underlying molecular mechanisms. With the increasing content of the gene-gene interaction databases, such as protein-protein interaction (PPI) databases and pathway databases, several methods have been developed to identify the responsive gene modules by finding an active sub-network in genome-wide gene networks (mostly PPI networks) [5-14]. The previous methods usually formulate the module identification task as an optimization problem: first, a module score evaluating the significance of differential expression [5-10] (a few methods also consider the gene-gene co-expression information in the objective function [11,12]) of any given gene sub-network is introduced as the objective function; then heuristic searching or exact computational methods (linear programming) are implemented to find the sub-networks optimizing the objective function. The obtained sub-networks are regarded as the responsive gene modules (see review in [13]). Related methods have been successfully applied for analyzing many physiological processes, such as type 2 diabetes [15], immunology [8], breast cancer metastasis [10] and drug response [5].

Here we presented a new formulation of the module identification task: a group of closely connected and co-expressed differentially expressed (DE) genes in genome-wide gene networks are regarded as the signatures of the underlying responsive gene modules at the RNA expression level. Our method named ClustEx was designed to find those signatures in the first step. Many studies show that the genes which are co-expressed in RNA level and/or interacted in protein level tend to involve in the same biological process, and promising new discoveries have been found by using the co-expression [16,17] and/or interaction information [18-20]. After getting the clustered DE genes as the signatures, the "missing parts" of the responsive gene modules are recovered in the second step by adding the intermediate genes, which may not be differentially expressed but are on the paths connecting the DE genes in the gene network.

Human umbilical vein endothelial cells (HUVECs) are widely used as *in vitro *models to study the vascular systems in inflammation and angiogenesis. We collected two time-course microarray datasets: one is for tumor necrosis factor alpha (TNF) stimulated HUVECs, an inflammation model [21-24], and the other one is for vascular endothelial growth factor A (VEGF) stimulated HUVECs, a canonical angiogenesis model [25-28]. Then ClustEx was applied to identify the responsive gene modules of TNF/VEGF stimulated HUVECs by integrating the time-course microarray data and the genome-wide HPRD PPI data [29-31]. Results show that ClustEx has better performances than several available module identification tools on the reference responsive gene sets. The enriched KEGG pathways [32], microRNA (miRNA) target gene sets [33,34] and GO terms [35] identified by gene set analysis also support ClustEx predictions.

## Results

### ClustEx overview: identify the responsive gene modules by network-based differentially expressed (DE) genes clustering and extending

**Figure 1:**
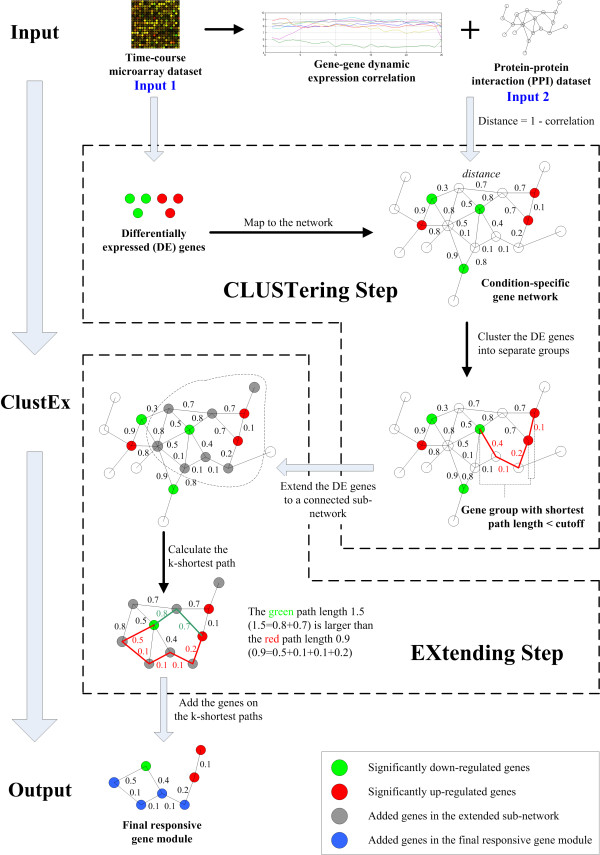
**The ClustEx workflow**. In the clustering step, DE genes were clustered and partitioned into relatively separate gene groups. In the extending step, intermediate genes on the k-shortest paths of each group of clustered DE gene were added to form the final responsive gene modules.

ClustEx is a two-step method for identifying the responsive gene modules by combining gene expression and interaction information. In the clustering step, average linkage hierarchical clustering was used to cluster and partition the DE genes into different gene groups according to their distances in gene networks, based on the assumption that a group of closely-connected and co-expressed DE genes are the signatures of the underlying responsive gene modules. In the extending step, the intermediate genes on the k-shortest paths between the DE genes were added to form the final responsive gene modules (Figure 1). The details of ClustEx are presented in Methods section.

### Identification of the responsive gene modules of human umbilical vein endothelial cells (HUVECs) in inflammation

**Figure 2:**
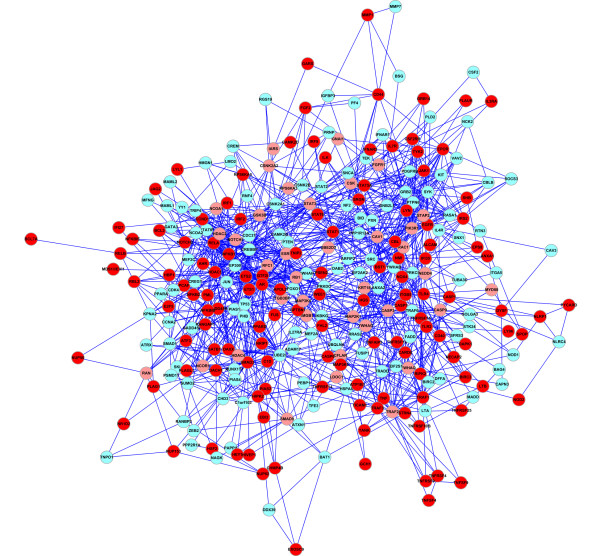
**The biggest responsive gene module of TNF stimulated HUVECs**. The "red" circles indicate the clustered DE genes. The "pink" circles indicate the intermediate genes on the shortest paths of the DE genes. The "light blue" circles indicate the intermediate genes on the 2-10 shortest paths of the DE genes.

ClustEx was applied to identified the responsive gene modules of HUVECs in inflammation model using the 0~8 h time-course microarray expression profiling data (GSE9055, 0~8 h, 25 time points [36,37]) and the HPRD genome-wide PPI data [29-31], with the following settings: the minimum fold changes of DE genes is 2, the shortest path length is shorter than 0.8 for clustering and the "k" is 10 for adding the intermediate genes on the k-shortest paths. The identified biggest responsive gene module has 284 genes including 130 DE genes (Figure 2, Additional file 1) and the second has 34 genes including 18 DE genes. The top two modules are very significant according to the edge-based module score measurement defined by [11] (*z-score *= 50.279 for the biggest module; *z-score *= 9.72 for the second module).

**Figure 3:**
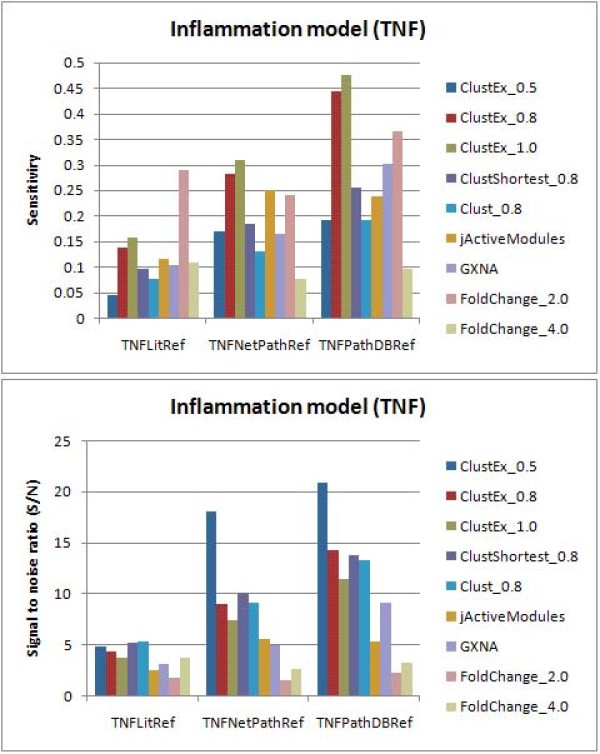
**The sensitivities and signal-to-noise ratios (S/N) for different computational methods on the TNF stimulated HUVECs dataset**. "ClustEx_0.5/0.8/1.0'' means the biggest module identified by ClustEx with distance cutoff 0.5/0.8/1.0, including 84/284/376 genes, respectively; "ClustShortest_0.8" means the biggest module identified by ClustEx only adding the intermediate genes on the shortest paths instead of the 10-shortest paths, including 167 genes; "Clust_0.8" means the biggest DE gene group identified by the clustering step, including 130 genes; "jActiveModules" means the top module identified by jActiveModules with greedy search [7], including 404 genes; "GXNA" means the highest scoring module identified by GXNA [8], including 300 genes; "FoldChange_2.0/4.0" means the DE genes with fold changes larger than 2.0/4.0, including 1421/260 genes.

To validate our predictions, three different TNF reference responsive gene sets were collected from 1) NetPath "TNF/NF-kB signaling pathway", 2) PID/BioCarta/Reactome annotated TNF signaling pathways, and 3) PubMed abstracts. We compared our predictions with several available module identification tools. The original node-based approach using simulated annealing (CytoScape jActiveModules plug-in [7]) and the edge-based heuristic searching approach in [11] (the Matlab and Java scripts were obtained by personal contact with the authors) did not find any significant module larger than 30 genes using the parameter settings described in Method section. The other compared methods included the node-based approach using greedy search (jActiveModules), GXNA (Gene eXpression Network Analysis) [8], several methods revised from ClustEx and the simple DE gene approach with minimum fold change (FoldChange_ [fold]) (Figure 3). Generally, ClustEx predictions are better both on sensitivity and signal-to-noise ratio (S/N) on the reference responsive gene sets, except that FoldChange_2.0 (with minimum fold change 2.0) exhibits much higher sensitivity on the literature reference gene set (TNFLitRef). As the cutoff of the hierarchical clustering is gradually relaxed (from 0.5 to 1.0), the sensitivity of ClustEx increases but the S/N decreases. The other two module identification methods also show higher specificities than FoldChange_2.0, which suggests that the interaction data of the gene network provide additional information of cell responses at the molecular level.

Gene set analysis of KEGG pathways, GO biological processes and microRNA (miRNA) target genes were conducted to find additional supporting evidence. Sixteen pathways were enriched in the biggest responsive gene module identified by ClustEx, including many known pathways affected by TNF, such as *Apoptosis*, *Notch signaling pathway*, *Jak-STAT signaling pathway*, *Toll-like receptor signaling pathway *and *Cell cycle *(Table 1, Additional file 2). Years ago, apoptosis in vascular endothelial cells has been reported after TNF stimulus [38,39]. Looking at the overlapped genes, it is found that caspase apoptosis cascade (CASP3, CASP6, CASP7 and CASP9 in the module) may be activated by TNF. *Jak-STAT signaling pathway *and *Toll-like receptor signaling pathway *are two signaling pathways activated by TNF [40-42]. Our previous study, which used another two micro-array datasets of TNF-stimulated vascular endothelial cells, also found that *apoptosis*, *Toll-like receptor signaling pathway *and *Jak-STAT signaling pathway *are enriched for the responsive process [43]. jActiveModules found eleven enriched pathways, GXNA found five pathways and FoldChange_2.0 found nine pathways. The average rank of the pathway enrichments was higher for ClustEx (average rank 1.86) than the other three methods (jActiveModules 2.32, GXNA 3.18, DE gene approach 2.64) (Table 1).

**Table 1:** The enriched pathways of the responsive gene modules of TNF stimulated HUVECs identified by different methods.

Pathway	ClustEx *Meet/Min (z-score)*	jActiveModules *Meet/Min (z-score)*	GXNA *Meet/Min (z-score)*	FoldChange_2.0 *Meet/Min (z-score)*
Apoptosis	**0.29(6.14), 1^a^**	**0.22(4.00), 3**	**0.15(3.70), 4**	**0.28(4.31), 2**
Adipocytokine signaling pathway	**0.27(4.88), 1**	0.15(1.39), 4	0.13(2.32), 3	**0.26(3.16), 2**
Prostate cancer	**0.24(4.83), 2**	**0.27(5.52), 1**	0.10(1.58), 3	0.16(0.96), 4
Notch signaling pathway	**0.33(4.78), 2**	0.18(1.58), 3	0.05(-0.24), 4	**0.38(4.80), 1**
Jak-STAT signaling pathway	**0.20(4.75), 1**	0.09(0.09), 4	0.07(0.83), 3	**0.21(3.38), 2**
Toll-like receptor signaling pathway	**0.23(4.68), 2**	**0.20(3.49), 3**	0.11(2.21), 4	**0.31(5.16), 1**
Small cell lung cancer	**0.24(4.58), 1**	**0.21(3.78), 2**	**0.14(3.42), 3**	0.21(2.57), 4
Huntington's disease	**0.32(4.12), 2**	**0.36(4.57), 1**	0.11(1.11), 3	0.14(0.25), 4
Chronic myeloid leukemia	**0.23(4.05), 3**	**0.27(5.55), 1**	**0.19(4.91), 2**	**0.26(3.71), 4**
Acute myeloid leukemia	**0.26(4.04), 1**	0.20(2.75), 2	0.13(2.32), 3	0.22(2.21), 4
Pancreatic cancer	**0.23(4.02), 2**	**0.23(4.28), 1**	0.10(1.40), 4	0.19(1.83), 3
Cell cycle	**0.19(3.52), 2**	**0.23(5.53), 1**	0.07(0.73), 4	0.15(0.74), 3
Neurodegenerative Diseases	**0.28(3.49), 1**	0.22(2.49), 2	0.03(-0.85), 4	0.17(0.73), 3
Epithelial cell signaling in Helicobacter pylori infection	**0.23(3.40), 2**	0.19(2.59), 4	**0.16(3.18), 3**	**0.28(3.49), 1**
Dorso-ventral axis formation	**0.29(3.17), 1**	0.25(2.61), 2	0.08(0.45), 4	0.25(1.85), 3
B cell receptor signaling pathway	**0.21(3.06), 1**	0.15(1.35), 4	0.11(1.82), 3	0.23(2.57), 2
Bladder cancer	0.23(2.85), 2	**0.26(3.47), 1**	0.13(1.70), 4	0.26(2.60), 3
T cell receptor signaling pathway	0.18(2.85), 3	0.09(-0.21), 4	**0.13(3.26), 1**	0.22(2.97), 2
Endometrial cancer	0.20(2.55), 2	**0.22(3.16), 1**	0.12(1.90), 3	0.12(-0.06), 4
Adherens junction	0.15(1.69), 2	**0.21(3.38), 1**	0.07(0.40), 3	0.11(-0.38), 4
Cytokine-cytokine receptor interaction	0.10(0.86), 3	0.05(-2.73), 4	0.08(1.25), 2	**0.22(4.76), 1**
TGF-beta signaling pathway	0.11(0.56), 4	0.15(1.83), 2	0.10(1.67), 3	**0.28(4.30), 1**

^a ^The rank of the pathway gene set enrichment of the compared methods according to the corresponding *Meet/Min *values' *z-scores*. The **bold font **denotes the enriched pathways (*z-score *> 3.0).

For the enriched miRNA target gene sets (the target gene sets are downloaded from the TargetScan website [33]): comparing with five for jActiveModules, four for GXNA and six for FoldChange_2.0, ClustEx found eight miRNAs, more than the other methods (Table 2, Additional file 3). These results suggest that ClustEx captures more signaling and regulatory information from the gene expression and interaction data of TNF stimulated HUVECs. In the enriched miRNAs, miR-221/222 is a well-studied miRNA which can significantly reduce tube formation and migration by directly targeting KIT (c-kit) [44,45]. In the identified biggest TNF responsive gene module, ETS1, IRF2, ESR1 and SOCS3, which are important genes in inflammation and angiogenesis, are also predicted as the targets of miR-221/222. MiR-18 is located in a large miRNA cluster miR-17~92, which has been identified as an oncogene [46]. It functions as a pro-angiogenic factor by repressing THBS1 (Tsp-1). MiR-18 is also predicted to target ESR1, IRF2, KIT, NOTCH2, PAPPA and TNFAIP3 in our study. MiR-145 has recently been reported to regulate cell differentiation [47,48]. A set of inflammatory and/or angiogenic genes, including ADAM17, CD40, ETS1, FOXO1, SMAD3 and TLR4, are predicted as the targets of miR-145, which suggests that miR-145 may also play important role in the two processes.

**Table 2:** The enriched miRNA target gene sets of the responsive gene modules of TNF stimulated HUVECs identified by different methods.

miRNA	ClustEx *tscore (z-score)*	jActiveModules *tscore (z-score)*	GXNA *tscore (z-score)*	FoldChange_2.0 *tscore (z-score)*
miR-216/216b	**33.49(3.48), 3^a^**	**24.09(2.85), 4**	**15.95(5.23), 1**	**31.43(3.62), 2**
miR-18ab	**30.18(3.16), 1**	**24.28(3.06), 2**	3.40(0.54), 4	17.73(1.33), 3
miR-145	**60.49(2.60), 3**	**53.82(3.01), 1**	**19.16(2.61), 2**	**53.52(2.22), 4**
miR-875-5p	**14.64(2.58), 1**	**10.46(2.03), 3**	0.00(-0.51), 4	**11.53(2.12), 2**
miR-7/7ab	**35.26(2.31), 1**	21.11(1.08), 2	4.85(0.50), 4	24.64(1.07), 3
miR-410	**46.88(2.29), 1**	34.59(1.73), 3	6.67(0.42), 4	**43.50(2.10), 2**
miR-221/222	**34.97(2.18), 2**	**29.68(2.21), 1**	3.88(0.14), 4	31.48(1.99), 3
miR-203	**53.31(2.18), 2**	38.25(1.47), 3	**19.65(3.03), 1**	40.47(1.02), 4
miR-143	28.69(1.85), 3	21.19(1.40), 4	**19.89(5.86), 1**	**39.81(3.84), 2**
miR-383	6.83(0.34), 3	3.03(-0.34), 4	2.34(0.71), 2	**14.23(2.24), 1**

^a ^The rank of the miRNA target gene set enrichment of the compared methods according to the corresponding *tscores*' *z-scores*. The **bold font **denotes the enriched miRNA target gene sets (*z-score *> 2.0).

We also analyzed the enriched GO terms of the biggest responsive gene module. The enriched terms for TNF are mainly divided into three classes: *apoptosis*, *protein kinase cascade *and *I-kB kinase/NF-kB cascade*. *Apoptosis *and *I-kB kinase/NF-kB cascade *are two main programs activated by TNF. These two GO terms are consistent with the enriched KEGG pathways. The detail information of the enriched GO terms is documented in Additional file 4.

### Identification of the responsive gene modules of HUVECs in angiogenesis

**Figure 4:**
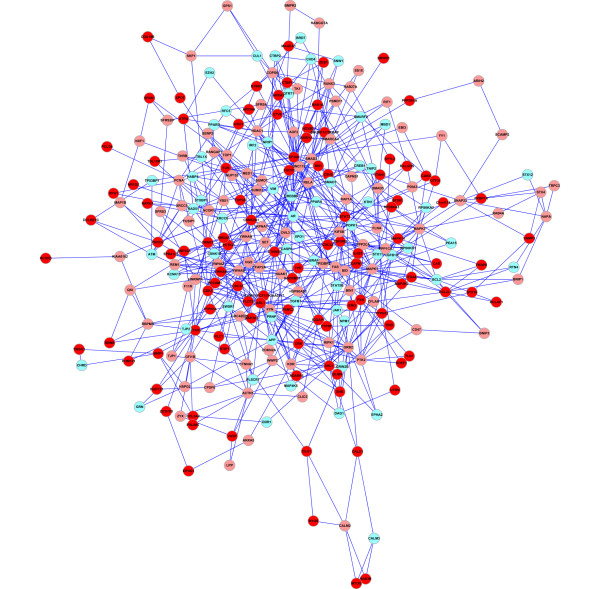
**The biggest responsive gene module of VEGF stimulated HUVECs**. The "red" circles indicate the clustered DE genes. The "pink" circles indicate the intermediate genes on the shortest paths of the DE genes. The "light blue" circles indicate the intermediate genes on the 2-10 shortest paths of the DE genes.

**Figure 5:**
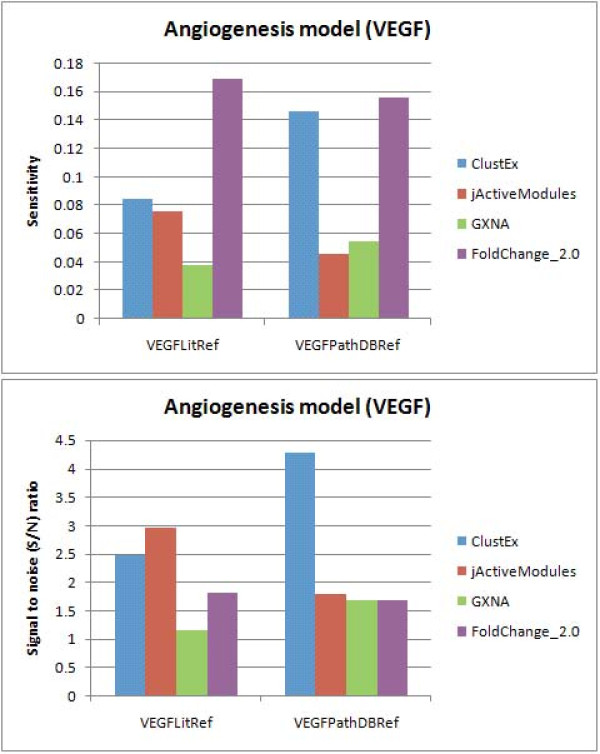
**The sensitivities and signal-to-noise ratios (S/N) for different computational methods on the VEGF stimulated HUVECs dataset**. "ClustEx" means the biggest module identified by ClustEx, including 262 genes; "jActiveModules" means the top module identified by jActiveModules with greedy search [7], including 195 genes; "GXNA" means the highest scoring module identified by GXNA [8], including 250 genes; "FoldChange_2.0" means the DE genes with fold changes larger than 2.0, including 709 genes.

Angiogenesis is an essential physiological process in vascular systems. ClustEx was applied to analyze a time-course microarray dataset of VEGF stimulated HUVECs (GSE10778, 0~6 h, 5 time points [49]), a canonical angiogenesis model [25-28]. The biggest responsive gene module has 262 genes, including 106 DE genes (Figure 4, Additional file 1). The *z-score *of the biggest module is 39.81. On the literature reference gene set (VEGFLitRef), FoldChange_2.0 achieves highest sensitivity and ClustEx show competitive performance with jActiveModules, while on the reference gene set collected from pathway databases (VEGFPathDBRef), ClustEx achieves highest specificity and competitive sensitivity to FoldChange_2.0 (Figure 5).

For the following gene set analysis: thirteen pathways and eight enriched miRNA target gene sets were found enriched in the biggest responsive gene module identified by ClustEx; nine pathways and eight miRNAs were found for jActiveModules; one pathway and six miRNAs were found for GXNA; and three pathways and six miRNAs were found for FoldChange_2.0 (Tables 3, Additional file 2 and Table 4, Additional file 3). In the enriched pathways, *TGF-beta signaling pathway*, *Cell cycle *and *Wnt signaling pathway *are frequently reported to be related to VEGF stimulus [50,51]. In the enriched miRNAs, miR-125 is detectable in HUVECs [52] and miR-200 has been reported to play an important role in angiogenesis and tumorigenesis [53]. MiR-132/212, ranked as the first for the VEGF dataset, may regulate angiogenesis by targeting EP300, MAP3K3, MAPK1 and MAPK3. The enriched GO biological processes are mainly *(anti-)apoptosis *and *RNA/nucleic acid transport *related terms (Additional file 4), which is consistent with VEGF pro-angiogenesis effect.

**Table 3:** The enriched pathways of the responsive gene modules of VEGF stimulated HUVECs identified by different methods.

Pathway	ClustEx *Meet/Min (z-score)*	jActiveModules *Meet/Min (z-score)*	GXNA *Meet/Min (z-score)*	FoldChange_2.0 *Meet/Min (z-score)*
Chronic myeloid leukemia	**0.25(6.20), 1^a^**	**0.19(5.32), 2**	0.07(0.72), 4	**0.19(3.67), 3**
TGF-beta signaling pathway	**0.23(5.88), 1**	**0.14(3.40), 2**	0.04(-0.53), 4	0.14(2.20), 3
Adherens junction	**0.22(4.74), 1**	0.10(1.73), 3	0.12(2.50), 2	0.12(1.23), 4
Pancreatic cancer	**0.19(3.99), 2**	**0.18(4.76), 1**	0.06(0.13), 4	0.17(2.76), 3
Neurodegenerative Diseases	**0.24(3.75), 1**	0.12(1.57), 2	0.03(-0.61), 4	0.12(0.81), 3
Focal adhesion	**0.13(3.66), 1**	0.08(1.72), 2	0.07(1.16), 4	0.11(1.71), 3
Cell cycle	**0.16(3.51), 2**	**0.16(5.02), 1**	0.01(-1.89), 4	0.05(-1.27), 3
Long-term potentiation	**0.19(3.47), 1**	0.10(1.52), 2	0.05(-0.05), 4	0.08(0.13), 3
Wnt signaling pathway	**0.14(3.24), 1**	0.06(0.35), 2	0.04(-0.74), 3	0.05(-1.03), 4
Prostate cancer	**0.16(3.15), 2**	**0.15(3.74), 1**	0.06(0.38), 4	0.10(0.72), 3
SNARE interactions in vesicular transport	**0.23(3.11), 1**	0.00(-1.43), 4	0.00(-1.38), 3	0.06(-0.31), 2
Renal cell carcinoma	**0.17(3.09), 1**	0.09(1.44), 3	0.08(0.93), 4	0.14(1.90), 2
Notch signaling pathway	**0.21(3.04), 2**	**0.27(5.60), 1**	0.03(-0.54), 4	0.12(0.89), 3
Acute myeloid leukemia	0.17(2.74), 2	**0.17(3.73), 1**	0.06(0.15), 4	0.13(1.43), 3
Endometrial cancer	0.17(2.47), 2	**0.17(3.42), 1**	0.06(0.29), 4	0.10(0.58), 3
Dorso-ventral axis formation	0.22(2.43), 2	**0.22(3.11), 1**	0.09(0.71), 3	0.09(0.06), 4
Apoptosis	0.13(1.95), 2	0.04(-0.77), 3	0.03(-1.19), 4	**0.19(3.63), 1**
Fc epsilon RI signaling pathway	0.09(0.57), 4	0.08(0.70), 3	**0.17(4.40), 1**	0.15(2.23), 2
Small cell lung cancer	0.06(-0.54), 3	0.11(2.27), 2	0.04(-0.70), 4	**0.20(4.31), 1**

^a ^The rank of the pathway gene set enrichment of the compared methods according to the corresponding *Meet/Min *values' *z-scores*. The **bold font **denotes the enriched pathways (*z-score *> 3.0).

**Table 4:** The enriched miRNA target gene sets of the responsive gene modules of VEGF stimulated HUVECs identified by different methods.

miRNA	ClustEx *tscore (z-score)*	jActiveModules *tscore (z-score)*	GXNA *tscore (z-score)*	FoldChange_2.0 *tscore (z-score)*
miR-132/212	**67.20(4.87), 1^a^**	**13.39(2.56), 3**	2.24(-0.23), 4	**28.71(2.78), 2**
miR-194	**58.17(3.89), 1**	3.51(-0.49), 4	2.41(-0.12), 3	23.27(1.79), 2
miR-216/216b	**38.29(3.29), 1**	**11.46(2.82), 3**	**7.40(2.95), 2**	13.48(1.35), 4
miR-328	**22.21(3.16), 1**	0.69(-0.43), 4	0.34(-0.37), 3	**10.79(2.09), 2**
miR-342/342-3p	**32.50(3.02), 3**	**12.48(3.47), 2**	2.35(0.42), 4	**23.72(4.06), 1**
miR-490/490-3p	**22.05(2.73), 2**	0.24(-0.71), 4	0.00(-0.61), 3	**17.34(3.76), 1**
miR-200bc/429	**103.98(2.64), 1**	24.58(1.50), 3	14.12(1.57), 2	47.05(1.20), 4
miR-125a-3p	**25.22(2.31), 1**	1.71(-0.31), 2	0.11(-0.69), 3	2.27(-0.83), 4
miR-874	24.42(1.99), 2	3.03(0.11), 3	0.42(-0.60), 4	**17.07(2.46), 1**
miR-204/211	55.42(1.96), 2	7.44(-0.18), 4	5.03(0.23), 3	**35.96(2.34), 1**
miR-186	58.74(1.95), 2	**19.21(2.22), 1**	4.49(-0.02), 4	28.68(1.08), 3
miR-377	37.24(1.26), 2	**19.87(3.55), 1**	1.52(-0.69), 4	17.59(0.47), 3
miR-410	46.73(1.19), 2	**22.02(2.93), 1**	4.37(-0.01), 4	26.31(0.97), 3
miR-22	30.06(0.67), 2	**18.02(3.14), 1**	2.88(-0.08), 4	15.22(0.24), 3
miR-155	21.64(0.21), 3	**12.02(2.11), 1**	1.16(-0.64), 4	14.76(0.56), 2
miR-374/374ab	33.48(-0.02), 3	7.79(-0.20), 4	**11.42(2.31), 1**	27.80(1.04), 2
miR-495/1192	40.09(-0.05), 4	13.12(0.56), 3	**14.26(2.70), 1**	29.97(0.68), 2
miR-590/590-3p	39.34(-0.66), 3	11.95(-0.10), 2	**19.89(3.63), 1**	20.92(-0.95), 4
miR-183	9.12(-1.17), 4	2.57(-0.75), 3	**9.89(2.92), 1**	8.83(-0.57), 2
miR-24	9.71(-1.63), 4	1.79(-1.29), 2	**9.39(2.01), 1**	7.61(-1.38), 3

^a ^The rank of the miRNA target gene set enrichment of the compared methods according to the corresponding *tscores*' *z-scores*. The **bold font **denotes the enriched miRNA target gene sets (*z-score *> 2.0).

## Discussion

### The cross-talk between inflammation and angiogenesis in *Notch signaling pathway*

Several studies have shown that endothelial cells are closely related to angiogenesis within an inflammatory environment [22,23]. *Notch signaling pathway *may play essential role in the cross-talk between inflammation and angiogenesis [25,54-57]. This pathway was found enriched both in TNF and VEGF responsive gene modules identified by ClustEx. Several repressing signals of notch signaling pathway were found after TNF stimulus, which can promote angiogenesis sprouting with the following VEGF stimulus [25,54]. Some transcription factors in the identified responsive gene modules, such as RELA (NF-kB), YY1 and SMAD3, which are the direct and highly co-expressed neighbors of the genes in KEGG annotated *Notch signaling pathway*, may also participate in the signaling.

### Limitation of the protein-protein interaction edges

Some cell adhesion molecules of HUVECs significantly up-regulated in inflammation, such as ICAM1, VCAM1 and SELE were not covered in the identified responsive gene modules. We manually checked the expression correlations between these genes with their neighbor genes and found that the correlations are relatively low. The promoters of the three genes contain multiple transcription factor binding sites of the NF-kB complex (NFKB1, RELA), which are significantly up-regulated by TNF stimulus and covered in the biggest TNF responsive gene module (the annotations of the promoters and the transcription factor binding sites are obtain from Transcriptional Regulatory Element Database, TRED [58,59]). These observations suggest that the missed responsive genes are more likely to connect with the biggest responsive module by transcriptional regulation rather than protein-protein interaction. So the missing edges representing the transcriptional regulations (and other types of interactions or regulations) should be added in future studies.

## Conclusions

Taking the closely-connected and co-expressed differentially expressed (DE) genes in condition-specific gene networks as the signatures of the underlying responsive gene modules provides a new strategy to solve the module identification problem. The responsive gene modules can be identified by finding the extended sub-networks from groups of clustered DE genes. Following this strategy, a two-step method named ClustEx was proposed and applied to identify the responsive gene modules of HUVECs within inflammation and angiogenesis. ClustEx shows better performances than several available module identification tools on reference responsive gene sets. The following gene set analysis of pathways and miRNA target genes also support ClustEx predictions.

## Methods

### Time-course microarray and genome-wide protein-protein interaction (PPI) data

Two time-course datasets were downloaded from NCBI GEO database [60,61]: GSE9055, Affymetrix Human Genome U133 Plus 2.0 Array (U133Plus2.0), HUVECs stimulated with 10 ng/mL TNF, 0-8 h, 25 time points [36,37] and GSE10778, U133A, HUVECs stimulated with 100 ng/mL VEGF, 0-6 h, 5 time points [49]. Original CEL format files were downloaded and then processed by dChip [62]. The probe signals were collapsed as gene expression signals by the mean value if multiple probes hit the same gene.

PPI data were downloaded from HPRD (Release 7) [29-31]. Only the genes both in the HPRD PPI dataset and the microarray platform were used in this study.

### ClustEx workflow

#### 1) Identification of the differentially expressed (DE) genes

First, the maximum fold change (according to non-log-transformed signals) respect to the 0 h00 m signal was calculated for each gene. Then the genes with minimum 2-fold changes (either up-regulated or down-regulated) were selected as the DE genes. We found 1421 DE genes (15.7%) in the TNF dataset and 709 DE genes (9.36%) in the VEGF dataset.

#### 2) Clustering step: cluster and partition the DE genes into different groups based on their distances in condition-specific gene networks

Cell responses to environmental stimuli are usually organized as relatively separate responsive gene modules. We clustered and partitioned the DE genes into different groups based on their interactions and their dynamic expression correlations. Each edge of the gene network derived from HPRD PPIs was weighted as

And the distance between two direct-interacting genes was defined as

The gene-gene distance was defined as the length of the shortest path between the two genes in the gene network. The shortest path length between any pair of DE genes was calculated using Dijkstra's algorithm. Then average linkage hierarchical clustering was used to cluster the DE genes according to the gene-gene distances. Distance cutoff was set to partition the DE gene into separate gene groups.

Hierarchical model analysis (HMA), a basic density-based clustering algorithm, is also used to cluster the DE genes. The detail description of this algorithm and the corresponding results are presented in (Additional file 5 and 6).

#### 3) Clustering step: select the cutoff for the hierarchical clustering of the DE genes

**Figure 6:**
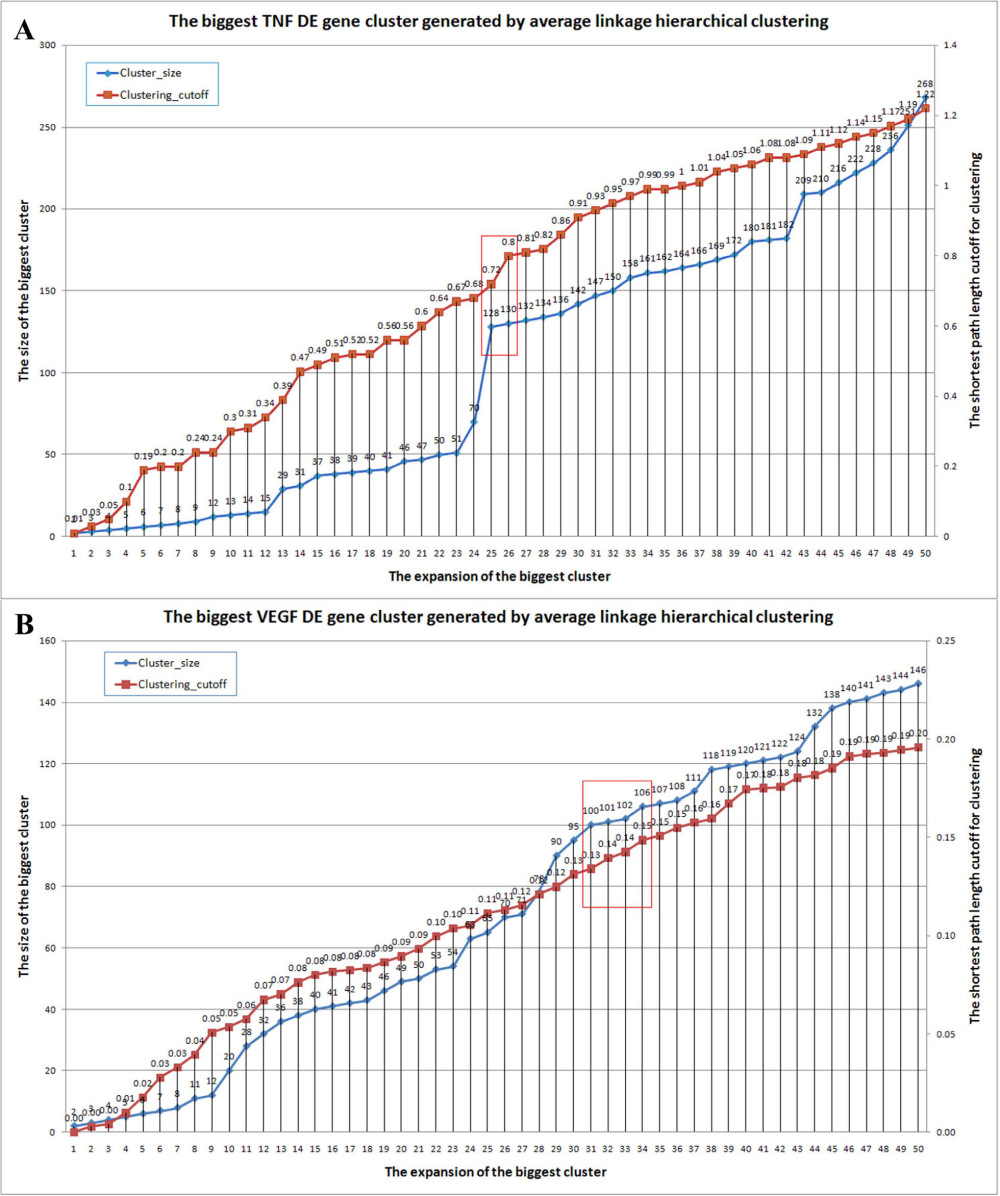
**The relationship between the hierarchical clustering cutoff and the size of the corresponding biggest DE gene group**.

As observed in previous studies and in our analysis, a big module usually "dominates" the responsive process [7,11]. We traced the size expansion of the biggest DE gene group and the increase of the corresponding distance cutoff. The cutoff is selected at the point after which the cluster expansion becomes much slower. For the TNF dataset, we observed a sharp turn right before 0.8 and the expansion of the cluster is much slower after 0.8 (Figure 6A), so we chose 0.8 as the cutoff to generate the DE gene clusters. For the VEGF dataset, a relative turn point exists around 0.14~0.15. We ran ClustEx with cutoff 0.14, 0.145, 0.15 and 0.155. The sizes of the final responsive gene modules are similar: 244, 247, 262 and 265, respectively. So we simply chose the cutoff at 0.15 (Figure 6B).

#### 4) Extending step: reconstruct the responsive gene modules by adding the intermediate genes connecting the DE genes

Microarray can detect the changes at the RNA expression level, but will miss many activity changes at protein level. It is assumed that the genes which are connecting the DE genes in the gene network are also important for cell responses. The final responsive gene modules were constructed by adding the intermediate genes to the DE gene groups found in the clustering step.

To reduce the false positives on the long paths and the huge computational cost for finding the k-shortest paths between all pairs of nodes in the whole gene network, the extending step was implemented as follows: first, the genes on the shortest paths between the DE genes were added to form a connected sub-network; then the sub-network was extended by one step in the whole gene network (it means the search space of the extending is limited in the DE genes, the genes on DE genes' shortest paths and the genes directly interacted with the former two kinds of genes); finally, the responsive gene modules were identified by extracting all the genes and edges on the 10-shortest paths between all the pairs of the DE genes in the extended sub-network. The k-shortest paths were calculated using an implementation of Yen's algorithm (k-shortest paths mean the shortest k [1^st^-k^th ^shortest] paths connecting the gene pair in the weighted network) [63]. Necessary changes were made in the source codes.

#### 5) Extending step: select "k" for the adding the genes on the k-shortest paths

**Figure 7:**
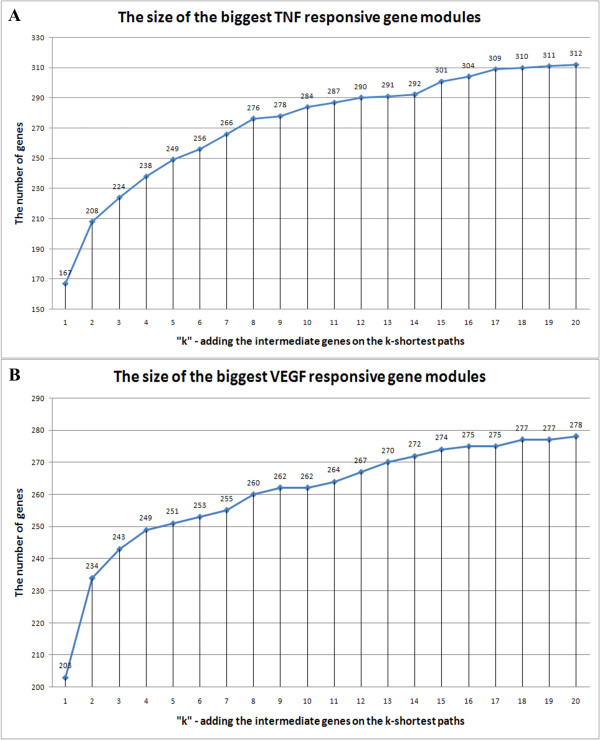
**The relationship between "k" and the size of the corresponding biggest responsive gene module**.

Similar to find the cutoff of the hierarchical clustering, we traced the size expansion of the biggest responsive gene module by increasing "k" from 1 to 20. No obvious cutoff was observed as in the curve of the size of the biggest DE gene cluster in the previous section. We empirically selected "k" as 10: the increased module size from 0 to 10 is more than 5 times as the increased size from 10 to 20 (for TNF dataset, 154/28 = 5.5; for VEGF dataset, 156/16 = 9.75) (Figure 7). The identified responsive gene modules are stable around the "k = 10": as the "k" reduces from 10 to 8, the size of the module is only reduced by 2.8% for the TNF dataset and by 0.8% for the VEGF dataset; as the "k" increases from 10 to 12, the size of the module is only increased by 2.1% for the TNF dataset and by 1.9% for the VEGF dataset. These small changes do little impact for the following analysis.

#### 6) Evaluate the statistical significance of the responsive gene modules

The evaluation method described in [11] was used to estimate the statistical significance of the identified responsive gene modules. First, the score for the edge connecting gene *x *and gene *y *was defined as

*sd*(*x*) and *sd*(*y*) are the standard derivations of the expressions of gene *x *and *y *in microarray datasets, respectively. |*cor*(*x*, *y*)| is the Pearson correlation of gene *x *and *y *(absolute value). The module score (*mscore*) was calculated by summing the *escores *of all edges in the module

Then we randomly sampled the same number of edges in the whole network and calculated the shuffled module score

The random sampling processes were repeated 10,000 times and the statistical significance was evaluated by *z-score*:

#### 7) ClustEx package for download

To facilitate the usage of ClustEx, we prepared the ClustEx package including two network distance calculation programs (modified Yen source codes are included in the package), several Perl scripts and the installation script. Users can download the package via our website: http://bioinfo.au.tsinghua.edu.cn/member/~gujin/clustex/ or via email: jgu@tsinghua.edu.cn. Current release requires huge computational cost, especially long waiting time. We will develop future version to solve this problem. We will also include the scripts to help determine the parameters of ClustEx (hierarchical clustering cutoff and "k" for the k-shortest path) in the future version.

### Evaluation of computational methods' performances by reference responsive gene sets

We prepared several reference responsive gene sets to evaluate the performances of the computational approaches:

**TNFLitRef **(TNF literature reference gene set), 376 genes. The gene symbols were analyzed and extracted from the 998 PubMed abstracts (before 2009/11/10) using keyword (TNF AND HUVEC*) by Agilent Literature Search (v2.71), a CytoScape plug-in. Then gene symbols were converted to Entrez Gene IDs by IDConverter [64] (a few genes not transferred by IDConverter were manually converted). The genes not covered by HPRD or Affy U133Plus2.0 array were removed. **TNFNetPathRef **(TNF NetPath pathway reference gene set), 184 genes. All Entrez Gene IDs were derived from "TNF signaling pathway" curated in NetPath database [65]. The genes not covered by HPRD or Affy U133Plus2.0 platform were removed. **TNFPathDBRef **(TNF pathway database reference gene set), 63 genes. Entrez Gene IDs of the reference genes were derived from following TNF related signaling pathways: BioCarta "TNF/stress related signaling", "TNFR1 signaling pathway and TNFR2 signaling pathway" [66], PID "TNF receptor signaling pathway" [67] and Reactome "TNF signaling" [68]. The genes not covered by HPRD or Affy U133Plus2.0 array were removed.

**VEGFLitRef **(VEGF literature reference gene set), 342 genes. The gene symbols were analyzed and extracted from the 871 PubMed abstracts (before 2009/11/10) using keyword (VEGF AND HUVEC*) by Agilent Literature Search (v2.71). Then gene symbols were converted to Entrez Gene IDs by IDConverter. The genes not covered by HPRD or Affy U133A array were removed. **VEGFPathDBRef **(VEGF pathway database reference gene set), 109 genes. Entrez Gene IDs of the reference genes were derived from BioCarta "VEGF, Hypoxia, and Angiogenesis", PID "Signaling events mediated by VEGFR1 and VEGFR2" and KEGG "VEGF signaling pathway" [32]. The genes not covered by HPRD or Affy U133A array were removed.

We compared the gene lists between the identified responsive gene modules and the reference gene sets. The sensitivity is defined as the percentage of genes in the reference gene set covered by the identified responsive gene module:

The signal-to-noise ratio (S/N) was used to evaluate the significance of overlapping. The signal is defined as the number of overlapped genes between the identified responsive gene module and the reference gene set; the noise is defined as the mean of the numbers of the overlapped genes between control modules and the reference gene set: 10,000 control gene sets each with the same size as the studied module were randomly sampling from the complete gene list and then *S/N *is calculated as the following definition:

### Comparison with other methods

jActiveModules with simulated annealing searching [7] and edge-base scoring method with simulated annealing searching (Matlab + Java codes were obtained by personal communication) [11] were run multiple times with different starting seeds and parameters, but neither one reported significant modules larger than 30 genes. Heuristic searching methods can find the (sub-)optimal results for the objective function if the iterations are long enough. But when the search space is bigger or the structure of the search space is irregular, the searching process is very slow. Due to the high computational cost, we may not be able to find the optimal parameter settings of these programs. Their predictions were not included in the comparison. For jActiveModules with greedy search, the top-scoring module was used in the comparison. EDGE software [69] was used to calculate the p-values evaluating the significances of gene expression changes in time-course microarray datasets, which were required as jActiveModules inputs. For Gene eXpression Network Analysis (GXNA) [8], the pre-defined sizes of the responsive gene modules were set as 300/250 genes for TNF/VEGF datasets. To fulfill GXNA input requirements, the 0 h00 m signals were repeated 24/4 times as control samples and the signals in the other 24/4 time points were used as case samples. Also due to the high computational cost, we may not be able to find the optimal parameter settings of these programs. The detail settings about the compared program were as follows:

a) The edge-based scoring method. The Matlab and Java codes are obtained by email. The package was run as the following parameters: simulated annealing start temperature 1 (default), end temperature 0.01 (default)/0.001 and iteration 30000 (default)/10000. The package was run multiple times with different random seeds. The produced biggest gene modules are no larger than 20 genes for the TNF dataset. Similar results are observed for the VEGF dataset.

b) jActiveModules with simulated annealing. This Cytoscape plug-in was run with the default parameter except changing the iteration to 100,000 (the parameter used in the original paper) and switching the Hubfinding On/Off. We ran multiple times with different random seeds. No significant modules were produced by the plug-in.

c) jActiveModules with greedy search. The program was run with its default parameter ("search depth" = 1 and "max depth from start node" = 2). The produced modules with the highest scores were used in the comparisons.

d) GXNA. The program was run with "-depth 300" for the TNF dataset (./gxna -name [tnf] -mapFile [tnf].ann -edgeFile [tnf].gra -algoType 1-version 001-depth 300) and "-depth 250" for the VEGF dataset (./gxna -name [vegf] -mapFile [vegf].ann -edgeFile [vegf].gra -algoType 1-version 001-depth 250).

### Gene set analysis of KEGG pathways, GO terms and miRNA target gene sets

*Meet/Min *values, commonly used to evaluate the overlapping of the two gene sets [70], were adapted to calculate the pathway/GO enrichments in the responsive gene modules. The GO terms with smaller than 50 genes and larger than 500 genes were removed. Larger *Meet/Min *values mean higher enrichments:

Degree preserving permutation methods were used to generate 1,000 random pathways and the *z-scores *of *Meet/Min *were calculated as:

The pathways with *z-score *> 3.0 were reported as enriched in the corresponding responsive gene modules.

Based on the assumption that the genes with higher expression changes, higher correlation with their neighbors and higher connection degrees would be more important, the network-based gene importance scores (*gscores*) were proposed to evaluate the importance of gene *x *in the responsive gene module:

To evaluate the enrichments of miRNA target gene sets, firstly the overlapped genes were found between the responsive gene modules and the miRNA target gene sets. Then the enrichments were calculated as the sums of the *gscores *of the overlapped target genes:

Degree preserving permutation methods were used to generate 1,000 random miRNA target gene sets and the *z-scores *of *tscores *were calculated as above. A looser cutoff was used to select enriched miRNA target gene sets (*z-score *> 2.0). TargetScan (v5.1) [33,34] miRNA target predictions were used in this analysis.

## Authors' contributions

GJ did most of the computational analyses and manuscript writings. CY focused on the biological background and the interpretation of the computational results. LS and LY lead the project and gave extensive instructions for this work. All authors read and approved the final manuscript.

## Supplementary Material

Additional file 1 **The biggest responsive gene modules**. The list of the Entrez IDs of the genes in the biggest responsive gene modules and the differentially expressed genes of TNF/VEGF stimulated HUVECs. Click here for file

Additional file 2 **The enriched KEGG pathways**. the detail results of the gene set analysis of KEGG pathways in the biggest responsive gene modules for TNF/VEGF stimuli. Click here for file

Additional file 3 **The enriched miRNA target gene sets**. the detail results of the gene set analysis of miRNA target gene sets in the biggest responsive gene modules for TNF/VEGF stimuli. Click here for file

Additional file 4 **The enriched GO terms**. The detail results of the gene set analysis of GO biological process terms in the biggest responsive gene modules for TNF/VEGF stimuli. Click here for file

Additional file 5 **The hierarchical mode analysis**. the description of the hierarchical mode analysis (HMA) algorithm and the corresponding results. Click here for file

Additional file 6 **The performance comparison among different module identification methods**. the detail results of the performance comparison among different methods, including ClustEx, ClustEx_HMA, jActiveModules and GXNA. Click here for file

## References

[B1] Hartwell LH Hopfield JJ Leibler S Murray AW From molecular to modular cell biology Nature 1999 402 C47 52 10.1038/35011540 10591225

[B2] Vidal M A biological atlas of functional maps Cell 2001 104 333 339 10.1016/S0092-8674(01)00221-5 11239391

[B3] Aderem A Systems biology: its practice and challenges Cell 2005 121 511 513 10.1016/j.cell.2005.04.020 15907465

[B4] Ravasz E Somera AL Mongru DA Oltvai ZN Barabasi AL Hierarchical organization of modularity in metabolic networks Science 2002 297 1551 1555 10.1126/science.1073374 12202830

[B5] Cabusora L Sutton E Fulmer A Forst CV Differential network expression during drug and stress response Bioinformatics 2005 21 2898 2905 10.1093/bioinformatics/bti440 15840709

[B6] Dittrich MT Klau GW Rosenwald A Dandekar T Muller T Identifying functional modules in protein-protein interaction networks: an integrated exact approach Bioinformatics 2008 24 i223 231 10.1093/bioinformatics/btn161 18586718 PMC2718639

[B7] Ideker T Ozier O Schwikowski B Siegel AF Discovering regulatory and signalling circuits in molecular interaction networks Bioinformatics 2002 18 Suppl 1 S233 240 12169552 10.1093/bioinformatics/18.suppl_1.s233

[B8] Nacu S Critchley-Thorne R Lee P Holmes S Gene expression network analysis and applications to immunology Bioinformatics 2007 23 850 858 10.1093/bioinformatics/btm019 17267429

[B9] Hwang T Park T Identification of differentially expressed subnetworks based on multivariate ANOVA BMC Bioinformatics 2009 10 128 10.1186/1471-2105-10-128 19405941 PMC2696448

[B10] Chuang HY Lee E Liu YT Lee D Ideker T Network-based classification of breast cancer metastasis Mol Syst Biol 2007 3 140 10.1038/msb4100180 17940530 PMC2063581

[B11] Guo Z Li Y Gong X Yao C Ma W Wang D Li Y Zhu J Zhang M Yang D Wang J Edge-based scoring and searching method for identifying condition-responsive protein-protein interaction sub-network Bioinformatics 2007 23 2121 2128 10.1093/bioinformatics/btm294 17545181

[B12] Zhao XM Wang RS Chen L Aihara K Uncovering signal transduction networks from high-throughput data by integer linear programming Nucleic Acids Res 2008 36 e48 10.1093/nar/gkn145 18411207 PMC2396433

[B13] Wu Z Zhao X Chen L Identifying responsive functional modules from protein-protein interaction network Mol Cells 2009 27 271 277 10.1007/s10059-009-0035-x 19326072

[B14] Maraziotis IA Dimitrakopoulou K Bezerianos A An in silico method for detecting overlapping functional modules from composite biological networks BMC Syst Biol 2008 2 93 10.1186/1752-0509-2-93 18976494 PMC2600641

[B15] Liu M Liberzon A Kong SW Lai WR Park PJ Kohane IS Kasif S Network-based analysis of affected biological processes in type 2 diabetes models PLoS Genet 2007 3 e96 10.1371/journal.pgen.0030096 17571924 PMC1904360

[B16] Lee HK Hsu AK Sajdak J Qin J Pavlidis P Coexpression analysis of human genes across many microarray data sets Genome Res 2004 14 1085 1094 10.1101/gr.1910904 15173114 PMC419787

[B17] Stuart JM Segal E Koller D Kim SK A gene-coexpression network for global discovery of conserved genetic modules Science 2003 302 249 255 10.1126/science.1087447 12934013

[B18] Stelzl U Worm U Lalowski M Haenig C Brembeck FH Goehler H Stroedicke M Zenkner M Schoenherr A Koeppen S A human protein-protein interaction network: a resource for annotating the proteome Cell 2005 122 957 968 10.1016/j.cell.2005.08.029 16169070

[B19] Bromberg KD Ma'ayan A Neves SR Iyengar R Design logic of a cannabinoid receptor signaling network that triggers neurite outgrowth Science 2008 320 903 909 10.1126/science.1152662 18487186 PMC2776723

[B20] Alexander RP Kim PM Emonet T Gerstein MB Understanding modularity in molecular networks requires dynamics Sci Signal 2009 2 pe44 10.1126/scisignal.281pe44 19638611 PMC4243459

[B21] Fiedler U Reiss Y Scharpfenecker M Grunow V Koidl S Thurston G Gale NW Witzenrath M Rosseau S Suttorp N Angiopoietin-2 sensitizes endothelial cells to TNF-alpha and has a crucial role in the induction of inflammation Nat Med 2006 12 235 239 10.1038/nm1351 16462802

[B22] Imhof BA Aurrand-Lions M Angiogenesis and inflammation face off Nat Med 2006 12 171 172 10.1038/nm0206-171 16462798

[B23] Pober JS Sessa WC Evolving functions of endothelial cells in inflammation Nat Rev Immunol 2007 7 803 815 10.1038/nri2171 17893694

[B24] Coussens LM Werb Z Inflammation and cancer Nature 2002 420 860 867 10.1038/nature01322 12490959 PMC2803035

[B25] Benedito R Roca C Sorensen I Adams S Gossler A Fruttiger M Adams RH The notch ligands Dll4 and Jagged1 have opposing effects on angiogenesis Cell 2009 137 1124 1135 10.1016/j.cell.2009.03.025 19524514

[B26] Hanahan D Folkman J Patterns and emerging mechanisms of the angiogenic switch during tumorigenesis Cell 1996 86 353 364 10.1016/S0092-8674(00)80108-7 8756718

[B27] Carmeliet P Jain RK Angiogenesis in cancer and other diseases Nature 2000 407 249 257 10.1038/35025220 11001068

[B28] Abdollahi A Schwager C Kleeff J Esposito I Domhan S Peschke P Hauser K Hahnfeldt P Hlatky L Debus J Transcriptional network governing the angiogenic switch in human pancreatic cancer Proc Natl Acad Sci USA 2007 104 12890 12895 10.1073/pnas.0705505104 17652168 PMC1931565

[B29] Peri S Navarro JD Amanchy R Kristiansen TZ Jonnalagadda CK Surendranath V Niranjan V Muthusamy B Gandhi TK Gronborg M Development of human protein reference database as an initial platform for approaching systems biology in humans Genome Res 2003 13 2363 2371 10.1101/gr.1680803 14525934 PMC403728

[B30] Keshava Prasad TS Goel R Kandasamy K Keerthikumar S Kumar S Mathivanan S Telikicherla D Raju R Shafreen B Venugopal A Human Protein Reference Database--2009 update Nucleic Acids Res 2009 37 D767 772 10.1093/nar/gkn892 18988627 PMC2686490

[B31] Mishra GR Suresh M Kumaran K Kannabiran N Suresh S Bala P Shivakumar K Anuradha N Reddy R Raghavan TM Human protein reference database--2006 update Nucleic Acids Res 2006 34 D411 414 10.1093/nar/gkj141 16381900 PMC1347503

[B32] KEGG http://www.genome.jp/kegg/

[B33] Lewis BP Burge CB Bartel DP Conserved seed pairing, often flanked by adenosines, indicates that thousands of human genes are microRNA targets Cell 2005 120 15 20 10.1016/j.cell.2004.12.035 15652477

[B34] Grimson A Farh KK Johnston WK Garrett-Engele P Lim LP Bartel DP MicroRNA targeting specificity in mammals: determinants beyond seed pairing Mol Cell 2007 27 91 105 10.1016/j.molcel.2007.06.017 17612493 PMC3800283

[B35] Ashburner M Ball CA Blake JA Botstein D Butler H Cherry JM Davis AP Dolinski K Dwight SS Eppig JT Gene ontology: tool for the unification of biology. The Gene Ontology Consortium Nat Genet 2000 25 25 29 10.1038/75556 10802651 PMC3037419

[B36] Kodama T Xu M Ohta Y Minami T Tsutsumi S Komura D Inoue K Kobayashi M Izumi A Miura M Time course gene expression of HUVEC after TNF-alpha treatment http://www.ncbi.nlm.nih.gov/projects/geo/query/acc.cgi?acc=GSE9055

[B37] Wada Y Ohta Y Xu M Tsutsumi S Minami T Inoue K Komura D Kitakami J Oshida N Papantonis A A wave of nascent transcription on activated human genes Proc Natl Acad Sci USA 2009 106 18357 18361 10.1073/pnas.0902573106 19826084 PMC2761237

[B38] Polunovsky VA Wendt CH Ingbar DH Peterson MS Bitterman PB Induction of endothelial cell apoptosis by TNF alpha: modulation by inhibitors of protein synthesis Exp Cell Res 1994 214 584 594 10.1006/excr.1994.1296 7925652

[B39] Robaye B Mosselmans R Fiers W Dumont JE Galand P Tumor necrosis factor induces apoptosis (programmed cell death) in normal endothelial cells in vitro Am J Pathol 1991 138 447 453 1992769 PMC1886201

[B40] Phulwani NK Esen N Syed MM Kielian T TLR2 expression in astrocytes is induced by TNF-alpha- and NF-kappa B-dependent pathways J Immunol 2008 181 3841 3849 18768838 10.4049/jimmunol.181.6.3841 PMC2649826

[B41] Syed MM Phulwani NK Kielian T Tumor necrosis factor-alpha (TNF-alpha) regulates Toll-like receptor 2 (TLR2) expression in microglia J Neurochem 2007 103 1461 1471 10.1111/j.1471-4159.2007.04838.x 17961202 PMC2423670

[B42] Guo D Dunbar JD Yang CH Pfeffer LM Donner DB Induction of Jak/STAT signaling by activation of the type 1 TNF receptor J Immunol 1998 160 2742 2750 9510175

[B43] Gu J Li S Chen Y Li Y Integrative Computational Identifications of the Signaling Pathway Network Related to TNF-alpha Stimulus in Vascular Endothelial Cells Bioinformatics, Systems Biology and Intelligent Computing, International Joint Conference on; Shanghai 2009 IEEE Computer Society 422 427 full_text

[B44] Kuehbacher A Urbich C Zeiher AM Dimmeler S Role of Dicer and Drosha for endothelial microRNA expression and angiogenesis Circ Res 2007 101 59 68 10.1161/CIRCRESAHA.107.153916 17540974

[B45] Poliseno L Tuccoli A Mariani L Evangelista M Citti L Woods K Mercatanti A Hammond S Rainaldi G MicroRNAs modulate the angiogenic properties of HUVECs Blood 2006 108 3068 3071 10.1182/blood-2006-01-012369 16849646

[B46] He L Thomson JM Hemann MT Hernando-Monge E Mu D Goodson S Powers S Cordon-Cardo C Lowe SW Hannon GJ Hammond SM A microRNA polycistron as a potential human oncogene Nature 2005 435 828 833 10.1038/nature03552 15944707 PMC4599349

[B47] Cordes KR Sheehy NT White MP Berry EC Morton SU Muth AN Lee TH Miano JM Ivey KN Srivastava D miR-145 and miR-143 regulate smooth muscle cell fate and plasticity Nature 2009 460 705 710 19578358 10.1038/nature08195 PMC2769203

[B48] Xu N Papagiannakopoulos T Pan G Thomson JA Kosik KS MicroRNA-145 regulates OCT4, SOX2, and KLF4 and represses pluripotency in human embryonic stem cells Cell 2009 137 647 658 10.1016/j.cell.2009.02.038 19409607

[B49] Schweighofer B Testori J Sturtzel C Sattler S Mayer H Wagner O Bilban M Hofer E The VEGF-induced transcriptional response comprises gene clusters at the crossroad of angiogenesis and inflammation Thromb Haemost 2009 102 544 554 19718476 10.1160/TH08-12-0830 PMC2886966

[B50] Phng LK Potente M Leslie JD Babbage J Nyqvist D Lobov I Ondr JK Rao S Lang RA Thurston G Gerhardt H Nrarp coordinates endothelial Notch and Wnt signaling to control vessel density in angiogenesis Dev Cell 2009 16 70 82 10.1016/j.devcel.2008.12.009 19154719 PMC8114544

[B51] Walshe TE Dole VS Maharaj AS Patten IS Wagner DD D'Amore PA Inhibition of VEGF or TGF-{beta} signaling activates endothelium and increases leukocyte rolling Arterioscler Thromb Vasc Biol 2009 29 1185 1192 10.1161/ATVBAHA.109.186742 19461051 PMC2775449

[B52] Heusschen R van Gink M Griffioen AW Thijssen VL MicroRNAs in the tumor endothelium: Novel controls on the angioregulatory switchboard Biochim Biophys Acta 2009 19782719 10.1016/j.bbcan.2009.09.005

[B53] Olson P Lu J Zhang H Shai A Chun MG Wang Y Libutti SK Nakakura EK Golub TR Hanahan D MicroRNA dynamics in the stages of tumorigenesis correlate with hallmark capabilities of cancer Genes Dev 2009 23 2152 2165 10.1101/gad.1820109 19759263 PMC2751988

[B54] Sainson RC Johnston DA Chu HC Holderfield MT Nakatsu MN Crampton SP Davis J Conn E Hughes CC TNF primes endothelial cells for angiogenic sprouting by inducing a tip cell phenotype Blood 2008 111 4997 5007 10.1182/blood-2007-08-108597 18337563 PMC2384130

[B55] Hellstrom M Phng LK Hofmann JJ Wallgard E Coultas L Lindblom P Alva J Nilsson AK Karlsson L Gaiano N Dll4 signalling through Notch1 regulates formation of tip cells during angiogenesis Nature 2007 445 776 780 10.1038/nature05571 17259973

[B56] Tammela T Zarkada G Wallgard E Murtomaki A Suchting S Wirzenius M Waltari M Hellstrom M Schomber T Peltonen R Blocking VEGFR-3 suppresses angiogenic sprouting and vascular network formation Nature 2008 454 656 660 10.1038/nature07083 18594512

[B57] Suchting S Freitas C le Noble F Benedito R Breant C Duarte A Eichmann A The Notch ligand Delta-like 4 negatively regulates endothelial tip cell formation and vessel branching Proc Natl Acad Sci USA 2007 104 3225 3230 10.1073/pnas.0611177104 17296941 PMC1805603

[B58] Jiang C Xuan Z Zhao F Zhang MQ TRED: a transcriptional regulatory element database, new entries and other development Nucleic Acids Res 2007 35 D137 140 10.1093/nar/gkl1041 17202159 PMC1899102

[B59] Zhao F Xuan Z Liu L Zhang MQ TRED: a Transcriptional Regulatory Element Database and a platform for in silico gene regulation studies Nucleic Acids Res 2005 33 D103 107 10.1093/nar/gki004 15608156 PMC539958

[B60] Barrett T Troup DB Wilhite SE Ledoux P Rudnev D Evangelista C Kim IF Soboleva A Tomashevsky M Edgar R NCBI GEO: mining tens of millions of expression profiles--database and tools update Nucleic Acids Res 2007 35 D760 765 10.1093/nar/gkl887 17099226 PMC1669752

[B61] Edgar R Domrachev M Lash AE Gene Expression Omnibus: NCBI gene expression and hybridization array data repository Nucleic Acids Res 2002 30 207 210 10.1093/nar/30.1.207 11752295 PMC99122

[B62] Li C Wong WH Model-based analysis of oligonucleotide arrays: expression index computation and outlier detection Proc Natl Acad Sci USA 2001 98 31 36 10.1073/pnas.011404098 11134512 PMC14539

[B63] An implementation of Yen's algorithm http://code.google.com/p/k-shortest-paths/

[B64] Alibes A Yankilevich P Canada A Diaz-Uriarte R IDconverter and IDClight: conversion and annotation of gene and protein IDs BMC Bioinformatics 2007 8 9 10.1186/1471-2105-8-9 17214880 PMC1779800

[B65] NetPath http://www.netpath.org/

[B66] BioCarta Pathways http://www.biocarta.com/genes/index.asp

[B67] Pathway Interaction Database http://pid.nci.nih.gov/

[B68] Reactome http://www.reactome.org/

[B69] Leek JT Monsen E Dabney AR Storey JD EDGE: extraction and analysis of differential gene expression Bioinformatics 2006 22 507 508 10.1093/bioinformatics/btk005 16357033

[B70] Shalgi R Lieber D Oren M Pilpel Y Global and local architecture of the mammalian microRNA-transcription factor regulatory network PLoS Comput Biol 2007 3 e131 10.1371/journal.pcbi.0030131 17630826 PMC1914371

