# Supplementary material for: Identification of responsive gene modules by network-based gene clustering and extending: application to inflammation and angiogenesis
Source: BMC Syst Biol. 2010 Apr 21;4:47. doi: 10.1186/1752-0509-4-47 (PMC2873318; doi:10.1186/1752-0509-4-47)
Supplement: Additional file 5 — The hierarchical mode analysis. the description of the hierarchical mode analysis (HMA) algorithm and the corresponding results. [file 1752-0509-4-47-S5.DOC]

In the main text, the average linkage hierarchical clustering was used to cluster the DE genes based on the distance matrix. In this supplementary method, we show the clustering results that are produced by a density-based clustering method, hierarchical model analysis (HMA). Density-based clustering is a kind of un-supervising clustering method, which can also cluster data according to their distance matrix (Ester et al. KDD 1996). HMA was run according to the procedure described in (Gupta et al. IEEE-ACM Trans Comput Biol Bioinform 2008):

“1) Using the distance matrix of the DE genes, determine the distances *d^n^(g)* from each point (gene) *g* to its *n*-th nearest point (we reported the results with *n* = 10).

2) Sort the distances *d^n^(g)* in ascending order. The smallest distance in *d^n^* is the distance between the “densest” point (gene) and its *n*-th nearest neighbors. This densest point forms the first cluster mode.

3) The next dense point is the point with the next smallest value in *d^n^*; *r* is set to this value. The algorithm takes one of three actions:

(i) the new point does not lie within *r* of another dense point, in which case it initializes a new cluster mode,

(ii) the point lies within *r* of dense points from one cluster only, and the point is added to this cluster, or

(iii) the point is within *r* of dense points from multiple clusters. In this case the clusters concerned are fused into one, and the point joins the fused cluster.

4) A note is kept of the nearest-neighbor distance between clusters. Whenever *r* exceeds the distance between two clusters, the two clusters merge into a single cluster.

5) Steps 3 and 4 are iterated until all points are clustered. Note that at the end of the *i*-th iteration, the *i* densest points have been assigned cluster labels.”

The clustering was terminated when the biggest cluster reaches the same size as the average linkage hierarchical clustering (130 for TNF dataset and 106 for VEGF dataset). The obtained clusters are largely overlapped, 79.23% for TNF dataset and 87.74% for VEGF dataset. And the final responsive gene modules identified by adding the intermediate genes on the k-shortest paths are also largely overlapped, 81.69% for the TNF dataset and 90.46% for the VEGF dataset. When testing on the reference responsive gene sets, ClustEx_HMA (the same operations with original ClustEx except using HMA to generate DE gene clusters) achieves comparable sensitivity and specificity with the original ClustEx method on the TNF dataset; while ClustEx_HMA achieves higher sensitivity and a bit higher specificity on the VEGF dataset.


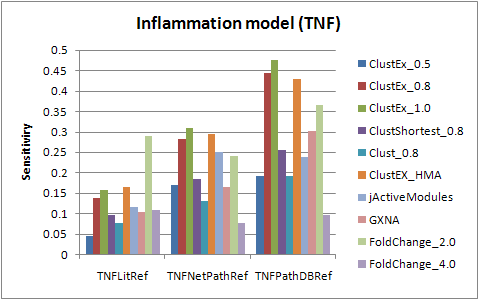


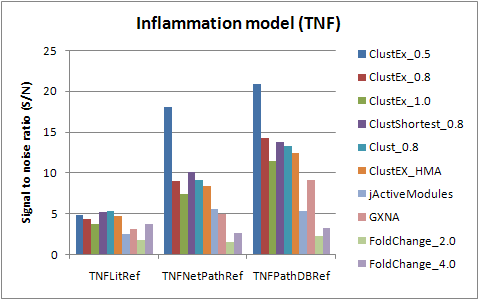


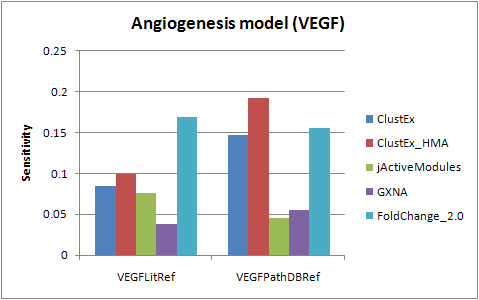


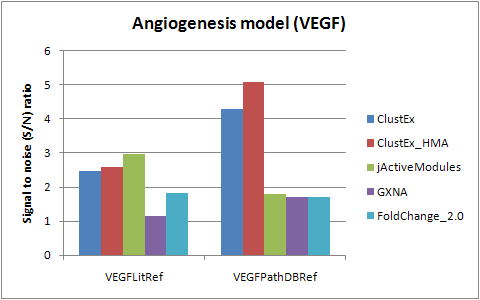


On these two datasets, HMA has a disadvantage that there is no turn point on the curve of the size expansion of the biggest cluster. It is hard to select a proper cutoff to generate the DE gene clusters. We kept the average linkage hierarchical clustering as the clustering algorithm in the clustering step of ClustEx. HMA is the simplest density-based clustering algorithm. We will test other advanced density-based clustering algorithms, such as DBSCAN and Auto-HDS for possible performance improvement.

**References**

Ester M, Kriegel H, Sander J, Xu X: **A density-based algorithm for discovering clusters in large spatial databases with noise**. In Proceedings of the 2nd International Conference on Knowledge Discovery and Data Mining (KDD-96), pages 226-231, Portland, OR, 1996.

Gupta G, Liu A, Ghosh J: **Automated Hierarchical Density Shaving: A robust, automated clustering and visualization framework for large biological datasets**. IEEE/ACM Trans Comput Biol and Bioinform 2008, (E pub).
